# Rubbing-Assisted Approach for Fabricating Oriented Nanobiomaterials

**DOI:** 10.3390/mi13081358

**Published:** 2022-08-20

**Authors:** Yadong Chai, Yanni Zhou, Motohiro Tagaya

**Affiliations:** 1 Department of Materials Science and Technology, Nagaoka University of Technology, Kamitomioka 1603-1, Nagaoka 940-2188, Japan; 2 Research Fellow of the Japan Society for the Promotion of Science (DC), 5-3-1 Koji-machi, Chiyoda-ku, Tokyo 102-0083, Japan

**Keywords:** biomimetic synthesis, biomaterials, orientation, rubbed polyimide, collagen, mesoporous silica

## Abstract

The highly-oriented structures in biological tissues play an important role in determining the functions of the tissues. In order to artificially fabricate oriented nanostructures similar to biological tissues, it is necessary to understand the oriented mechanism and invent the techniques for controlling the oriented structure of nanobiomaterials. In this review, the oriented structures in biological tissues were reviewed and the techniques for producing highly-oriented nanobiomaterials by imitating the oriented organic/inorganic nanocomposite mechanism of the biological tissues were summarized. In particular, we introduce a fabrication technology for the highly-oriented structure of nanobiomaterials on the surface of a rubbed polyimide film that has physicochemical anisotropy in order to further form the highly-oriented organic/inorganic nanocomposite structures based on interface interaction. This is an effective technology to fabricate one-directional nanobiomaterials by a biomimetic process, indicating the potential for wide application in the biomedical field.

## 1. Introduction

**Figure 1:**
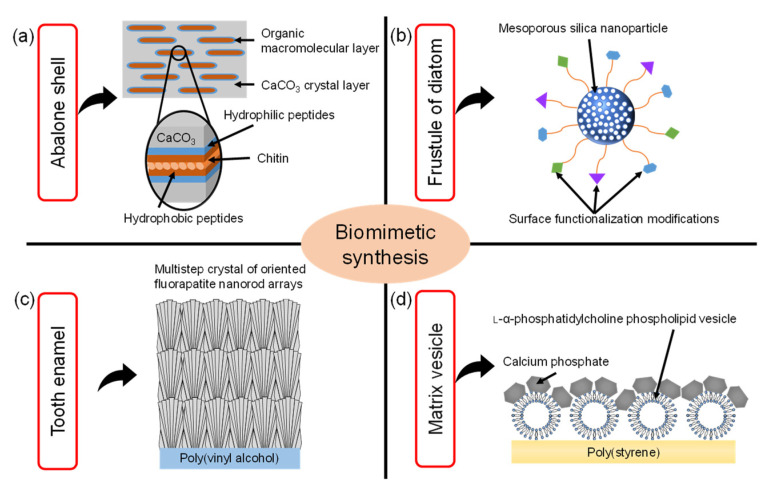
Illustration of the various biomimetic syntheses and their structures inspired by (**a**) nacre of the abalone shell, (**b**) frustule of diatom, (**c**) tooth enamel, (**d**) matrix vesicle shell (Reprinted with permission from Ref. [16]. ©2018, The Japan Society of Vacuum and Surface Science Publication).

Biomimetics is the field of science that aims to imitate the excellent functions and shapes of biological tissues to synthesize materials and apply them to engineering and medical fields. For example, as shown in Figure 1a, it is well known that nacre of abalone is a type of hybrid material consisting of calcium carbonate (CaCO_3_) and protein matrix [1]. The hierarchical structures in the nacre of abalone consist of a layered plate-like aggregate of nanocrystalline aragonite which is glued by organic matrix layers. The exoskeleton of crayfish also has a similar hybrid structure which mainly consists of α-chitin/protein microfibril frameworks and amorphous CaCO_3_ [2]. Inspired by these special structures in biomineralization, the synthetic technology of CaCO_3_/polymer hybrids has been developed. In the process of biomimetic synthesis of CaCO_3_, the polymer films such as chitin [3], chitosan [4] and poly(vinyl alcohol) [5] were used as insoluble organic templates. The CaCO_3_ films were formed on the organic templates with the soluble additives of acidic polymers such as poly(acrylic acid), poly(aspartic acid), and poly(glutamic acid). Then, acidic macromolecules caused the aggregation of Ca^2+^, leading to the nucleation of CaCO_3_. As a result, these thin CaCO_3_ crystalline films were composed of assembled nanocrystals with radially oriented *c* -axis.

The frustule of diatom is composed of amorphous porous silica [6,7,8]. The frustule consists of areolae which are honeycombs of hexagonal chambers. One side of the areola is covered by one or two dense porous membranes (cribrum and cribellum) [6]. Based on this structure, the porous silica nanostructures were widely researched. in particular, mesoporous silica (MPS) materials were synthesized through a sol–gel process where surfactants acting as structure-directing agents were used to produce mesoporous structures [9,10,11]. The MPS materials also have regularly arranged pores (size range of 2~50 nm) with a uniform diameter, large specific surface area (up to 1000 m^2^/g), pore volume (>0.9 cm^3^/g), and their surface can be easily functionalized with various functional groups, such as organic and metal species to enhance their biocompatibility [12], as shown in Figure 1b.

Tooth enamel is a highly mineralized tissue in the body where a very hard, thin, translucent layer of calcified tissue covers the entire anatomic crown of the tooth [13]. Tooth enamel is very hard because it is roughly composed of 95~98% inorganic materials mainly contained in apatite crystals. These apatite crystals contain trace minerals such as lead, fluoride, strontium and magnesium. The enamel of teeth is covered with organized slender apatite nanocrystals which are composed of highly-oriented bundles parallel to the *c*-axis of the nanocrystals. Artificial enamel-like apatite crystals have been widely applied for dental applications. For example, as shown in Figure 1c, enamel-like fluorapatite (FA) crystals were prepared on a poly(vinyl alcohol) (PVA) sheet by immersion in simulated body fluid containing fluoride ions [14]. The FA crystals obtained with multistep crystal growth on the PVA sheet were thicker than 100 μm. The high-density nucleation of FA crystals was caused by the interaction between the relatively high concentration of inorganic ions in simulated body fluid and abundant OH groups on the PVA sheet surface. Subsequently, the crystal growth of FA nanorods grew along the *c*-axis direction in simulated body fluid with a low concentration of inorganic ions. Using this multistep epitaxial growth method, the enamel-like crystals composed of *c*-axis oriented nanorods were obtained with diameters below 300 nm.

Matrix vesicles play an indispensable role in the process of biomineralization. In detail, osteoblasts secrete extracellular matrix vesicles equipped with a variety of membrane transporters and enzymes, which are necessary for the initial nucleation and subsequent growth of calcium phosphate crystals. Inspired by the biomineralization process of matrix vesicles, the unique calcium phosphate (CP)/L-α-phosphatidylcholine phospholipid vesicle (PV) hybrid film was developed [15]. As shown in Figure 1d, the CP hybridized with PV was formed on poly(styrene) tissue cultures which have transparency, unique structures, and good stability against sterilization treatments. Moreover, the osteoblasts cultured on the CP/PV hybrid films exhibited high osteogenic activities [16].

In order to further understand the mechanisms of biomineralization and crystal deposition, the nucleation and growth of inorganic compounds and biomimetic synthesis of inorganic/organic composite in gel systems have been researched [17,18,19,20,21,22,23,24]. Several gel systems have been explored including gelatin hydrogel, iota-carrageenan gel, starch gel, etc. For example, a method for biomimetic synthesis of CP crystals through single and double diffusion techniques in gel systems has been reported [19,20,21,22,23,24]. The morphology of synthesized CP crystals depends on the pH value and concentration of gel, ion concentration and diffusion type [20,21,22]. The nucleation and growth of brushite and octacalcium phosphate crystals were controlled in an iota-carrageenan gel and showed different morphologies [20]. The brushite crystals changed from highly porous aggregates to plate-shaped forms. The octacalcium phosphate crystals showed a porous spherical shape different from brushite growth forms. It is possible to control the CP crystallization effectively using this diffusion technique. Moreover, the brushite and octacalcium phosphate crystals were precipitated in the starch gel through the single and double diffusion techniques, resulting in the formation of plate-shaped and needle-like crystals [21]. The synthesized CP crystals show similar morphology to brushite kidney stones, which is useful for understanding the mechanism of kidney stone formation. In addition, the brushite crystals were synthesized through the single diffusion technique in gelatin hydrogel [22]. The brushite crystals were grown and showed the different morphologies in the gelatin hydrogel system according to the additives used such as glutamic acid and urea. The interactions among carboxylic groups of glutamic acid, amine groups of gelatin, and urea reduced the inclusion of calcium ions in brushite crystals, suggesting that glutamic acid-rich foods have the potential to inhibit and control brushite kidney stones. Interestingly, calcium iodate/gelatin composite particles [23] and zinc phosphate nanosheets [24] with antibacterial properties can also be synthesized using this gel diffusion technique and showed the alignment of nanostructures in one dimension. This gel diffusion technique is expected to develop novel functional materials for biomedical applications.

In this review, the oriented structures in biological tissues were reviewed and the techniques for producing highly-oriented biomaterials by imitating the oriented organic/inorganic nanocomposite mechanism of the biological tissues were summarized. In particular, we introduce the fabrication technology of the highly-oriented structure of biomaterials on the surface of a rubbed polyimide film that has physicochemical anisotropy in order to further form the highly-oriented organic/inorganic nanocomposite structures based on interface interaction. Moreover, we mention the possibility that the highly-oriented nanobiomaterials fabricated by the rubbing-assisted approach could be widely applied in the biomedical field.

## 2. Oriented Structures in Biological Tissues

### 2.1. Characteristic Structures in Cornea and Bone

**Figure 2:**
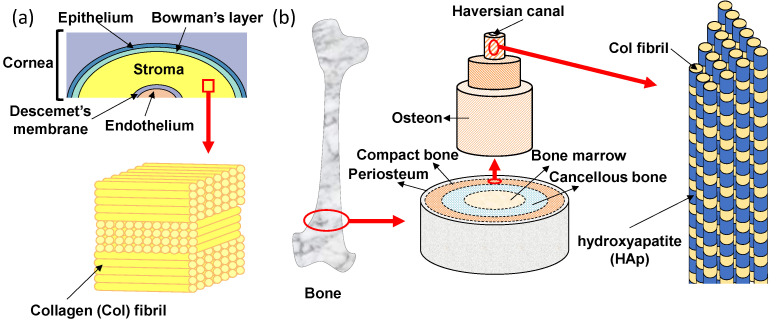
Oriented tissue Col structures of (**a**) human cornea and (**b**) bone.

The transparent cornea is one of the layered tissues in our eye and is located closest to the outside, covering the iris, pupil, and anterior chamber. In detail, as shown in Figure 2a, the layers of cornea are made up of five different membranous tissues which, from the outer layer to the inner layer, are the epithelium, Bowman’s layer, stroma, Descemet’s membrane and endothelium [25]. The stroma with a thickness of 500 μm exists between the epithelial and endothelial, which supports the strength of the cornea [26]. The stroma occupies more than 90% of the thickness of the cornea and plays a very important role in keeping the cornea transparent. The corneal stroma consists of more than 300 stacked lamellae rotated alternately by 90°. Each lamella is about 1~2 µm in thickness and contains tightly packed collagen fibrils that align in one direction [27,28]. This stacked lamella structure has two excellent functions. One is mechanical strength. Tensile strength is high because of the stacked lamellae in which collagen fibrils are arranged and stacked orthogonally. The other function is transparency. The transparency of the cornea is highly dependent on the uniform diameter and the spacing of the collagen fibrils, which are closely packed in a regular array so that the light passes only in a specific direction [29]. Moreover, the human stroma is formed in the mother’s body. Collagen fibrils in the corneal stroma can be replaced due to metabolism, but once the corneal stroma is damaged, it cannot be generated anew. Thus, all layers of the corneal epithelium, parenchyma, and endothelium must be transplanted when the cornea is damaged. If the lamellar structure of collagen fibrils can be formed in vitro, it will be useful for reconstruction of the stroma.

The bone also has an oriented structure of collagen fibrils that resembles the stroma of the cornea. The hierarchical structure of bone is shown in Figure 2b. Macroscopically, from the outside to the inside, bone consists of periosteum, compact bone, cancellous bone and bone marrow tissues [30]. On the outermost side, the periosteum consists of dense irregular connective tissue. On the inside of the periosteum, it is a very hard compact bone that supports our body. The cancellous bone, also called trabecular or spongy bone, is lighter and less dense than compact bone, which is a porous network organized by trabeculae. The bone marrow is a source of mesenchymal stem cells which can differentiate into various cells such as bone, cartilage, muscle, and fat. Bone gradient of mechanical strength goes from hard compact bone to soft bone marrow. In the compact bone, the bone unit called osteon can be seen [31]. In addition, a small tube called the Haversian canal is connected up and down in the middle of the bone unit, and is a pathway for supplying nutrients to the bone cells existing inside the bone. The osteon has an annual ring-like structure with several stratified lamellae around the Haversian canal [32]. There are regularly arranged collagen fibrils in each of the lamellae which also align in one direction. Collagen fibrils are arranged vertically between adjacent lamellae to the alternating laminated structure, so that the bone unit is flexible and has strong tensile and bending strength. In the osteon, there are hydroxyapatite (HAp) nanocrystals (with a size of 20−40 nm) parallel to the collagen fibril alignment direction [33]. The nucleation and growth of HAp crystals are regulated by collagen fibrils. The polarity of collagen is considered to be an important factor affecting bone mineralization [34,35]. It has been reported that the highly-oriented collagen fibrils have a dipole change corresponding to the change from N-terminus to C-terminus of the constituent collagen molecules, suggesting that the piezoelectricity of bone is likely due to the presence of collagen [36,37,38]. Recently, the precipitation of HAp has been shown to occurr on the collagen fibrils of bone tissues, suggesting that piezoelectric generation of electric charge may be a primary mechanism of bone remodeling [39]. A resonance-enhanced piezoresponse force microscopy (PFM) was utilized to evaluate the weak piezoelectric response of individual collagen fibrils [34]. The result indicated that the shear piezoelectric coefficient (*d*_15_) varied periodically along the collagen fibrils, with larger values in the hole zone (0.51 pm/V) compared to the overlap zone (0.29 pm/V). The higher piezoelectricity in the gap region can locally modulate the surface potential of collagen fibrils and further prompt the mineralization starting from the hole zone [40].

### 2.2. Proteins for Inorganic Mineral Precipitation and Orientation, and the Interactive Mechanism

**Figure 3:**
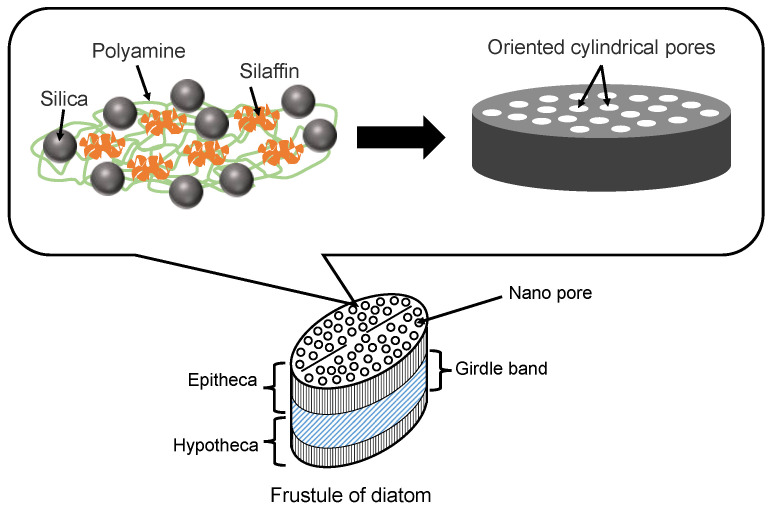
Illustration of diatom frustule structure and diatom proteins for silica precipitation.

Diatoms are mainly composed of small amounts of proteins (e.g., silaffins), polyamines, and amorphous hydrated silica [41]. Silaffins and polyamines are bound by electrostatic interactions between negatively charged groups of phosphate, sulfate, and carbohydrate (silaffins) and positively charged groups of amines (polyamines). Orthosilicic acid (Si(OH)_4_) is used to form silicon dioxide and is generally present in the environment at concentrations ranging from tens to hundreds of micromol per liter. Orthosilicic acid is transported to the cells by silicon transporter proteins. The orthosilicic acid interacts with special matrix peptides and proteins (e.g., silaffins, silacidins) and turns to amorphous hydrated silicon dioxide in the cells. Although there are many species of diatom frustules, their structures have some common characteristics. As shown in Figure 3, the main structure of a diatom frustule is composed of epitheca, a girdle band and hypotheca. Frustules have many micro- or nano-scale substructures [6,8,41], including multilevel pores, marginal processes, spines, and raphe. The multilevel pores are the main substructures. Circular or hexagonal chambers called areolae exist in the epitheca and hypotheca, which is composed of hundreds of large pores regularly arrayed. There are many pores with different diameters in the range of 40~200 nm in the areolae. In addition, the girdle band also has myriads of pores with a diameter of 100 nm.

### 2.3. Inorganic Minerals for Orientational Protein Adsorption, and the Interactive Mechanism

Many factors could affect protein adsorption. As a crude generalization, protein adsorption is mainly determined by the properties of the proteins, the properties of the substrates, the protein–substrate interactions, and the media [42]. In detail, many influencing factors should be considered. For example, the isoelectric point of the proteins, the surface potential of substrates, electrostatic interactions between proteins and substrates, pH of media, etc. In addition, the size-selective adsorption of proteins on porous material substrates is also an important factor [43].

Implant therapy creates an implant-tissue interface that is always exposed to the air resulting in the possibility of inflammation. Titanium as an important implant material has a greater ability than other metals to facilitate osseointegration [44,45,46]. The titanium oxide film formed on the titanium surface is one of the reasons for its high level of corrosion resistance. In addition, the degree of the deposition of calcium phosphates in body fluid is greater on titanium than on other metals [47,48,49]. When titanium is implanted into bone tissues, the adsorption of osteogenic proteins such as osteocalcin and osteopontin on to the titanium surface occurs. Titanium oxide has a similar isoelectric point to osteogenic proteins at approximately pH = 5. Accordingly, both titanium oxide and osteogenic proteins are negatively charged at around pH = 7. Thus, the deposition mechanism of calcium phosphates is readily caused by the positively charged calcium ions (Ca^2+^) and further adsorption of proteins is prompted on the deposited calcium phosphates. Moreover, the hydration effect of terminal OH radicals with positive charges is also considered to affect protein adsorption. Brunette et al. discuss how proteins attached to the arranged grooved surface prompt an osteogenic cell to differentiate into an osteoblast [50]. Therefore, it is necessary to develop an oriented biocompatible material which can control the orientation of adsorbed proteins and cells so as to further encourage the regeneration of biological tissue.

## 3. Oriented Collagen Molecular Assembly for Hydroxyapatite Composites

### 3.1. Assembly Structures

Collagen is abundantly present in biological tissues such as bone [51], cartilage [52], ligaments [53], tendons [54], stroma [27], skin [55], liver [56], and muscle [57], and accounts for about 30% of all proteins contained in organs. There are many types of collagen in the human body from type I to type XII. Type I collagen is mainly contained in the skin and bone and is also found in many parts of the body. Type II collagen mainly exists in cartilage, and Type III collagen often coexists in the tissues in which Type I collagen is present. Each type of collagen is present in several different organs. 

The type I collagen (Col) exists in many different biological tissues, and is the most abundant protein [58,59,60,61]. The Col molecule is composed of three polypeptide chains containing two α_1_ and one α_2_ polypeptide chains [62,63]. Each polypeptide chain has a repeating structure of three amino acids “G-X-Y”, including about 3000 amino acids. Here, G is glycine, which is the smallest of all amino acids. X is proline, and the remaining Y is occupied by various other amino acids, mainly hydroxyproline. Amino acids contained in the Col molecule can be roughly divided into polar and non-polar depending on the nature of the side chain (R). Polar amino acids are hydroxyproline, aspartic acid, threonine, serine, glutamic acid, cysteine, tyrosine, hydroxylysine, lysine, histidine, and arginine. Non-polar amino acids are proline, glycine, alanine, valine, methionine, isoleucine, leucine, and phenylalanine. The denaturation temperature of the Col molecule depends on the composition of amino acids and is in the range of several °C to 40 °C [64,65,66]. When the temperature is raised over the denaturation temperature, the triple helix structure of Col unravels into individual polypeptide chains leading to the formation of gelatin. The change in molecular structure from Col to gelatin is called denaturation. In addition, once the triple helix structure is completely unwound, it cannot return to the original triple helix structure even if the temperature is lowered. The denaturation temperature of Col varies depending on the species (that is, on the composition of amino acids). For example, the Col denaturation temperature of mammals is approximately 40 °C. In the case of fish, the Col denaturation temperature of tilapia is 30~36 °C [67,68,69].

**Figure 4:**
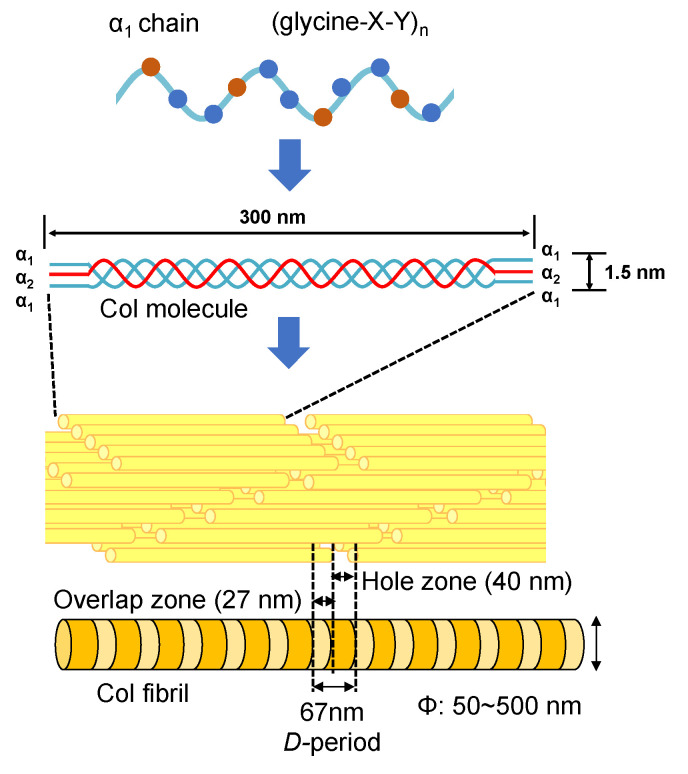
The structures of Col molecules and their various aggregation states.

As the smallest Col molecular assembly in biological tissues, Col fibrils play an important role. As shown in Figure 4, an axial periodicity (*D*-period) of approximately 67 nm has been reported in Col fibril structures, where hole zone and overlap zone were presented in *D*-period due to the self-assembled Col molecules [70,71]. The Col fibrils have uniform diameters in different biological tissues (corneal stroma (*ca.* 30 nm) [72,73], stapedius tendon (*ca.* 50 nm) [74] and compact bone (*ca.* 100 nm)) [75,76]. In addition, Col fiber (*ca.* 5~10 μm) with a larger diameter can be further assembled from Col fibrils [54]. The Achilles tendon is made of Col fibers and has strong rigidity in a certain direction. Moreover, the skin consists of a fiber bundle in which Col fibers are entwined [55].

### 3.2. Orientation Control of Collagen Assembly

Col assembly such as in Col fibrils is an important protein state that has various functions including skeletal support [77], cell adhesion [78], and guiding tissue regeneration [79]. Col assembly as a biomaterial is indispensable in regenerative medicine. As mentioned above, Col assembly with uniform diameter widely exists in different biological tissues and provides different functionality. The orientational Col assembly contributes to the orientational adsorption of proteins and the orientational differentiation and growth of cells. Thus, it is necessary to synthesize the oriented Col assembly similar to human tissues. The orientation control of the Col fibrils has been widely reported by magnetic field [80], microfluidic channel [81], and electrochemical methods [82]. However, it was found in these reports that the formed Col fibrils had low orientation (less than 50%) and non-uniform diameter (in the broad range from 50 to 200 nm), indicating that the orientation and uniformity of the Col fibrils have not been successfully controlled. Therefore, it is necessary to develop a technique for efficiently forming highly-oriented Col fibrils with uniform diameter.

### 3.3. Hydroxyapatite

**Figure 5:**
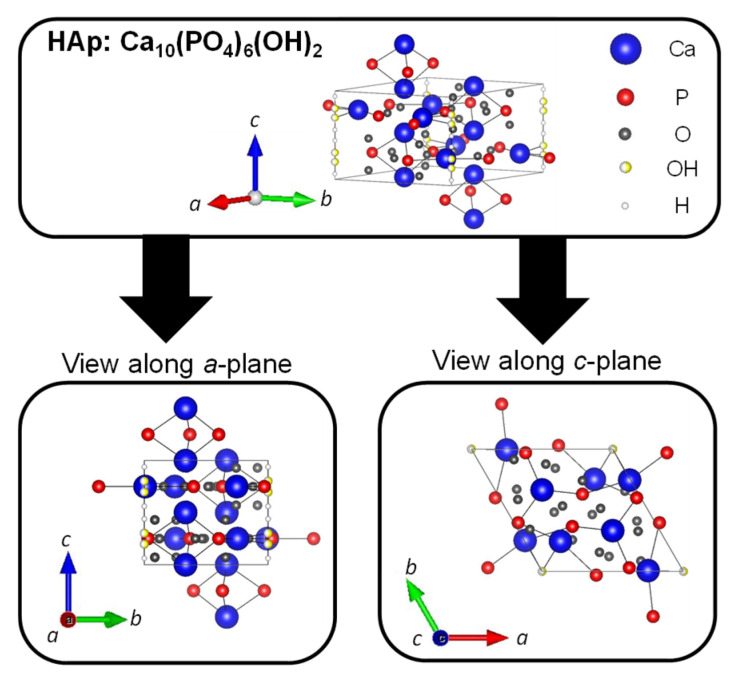
Crystal structures of HAp were drawn using VESTA from a crystallographic information file obtained from the American Mineralogist Crystal Structure Database.

Hydroxyapatite (HAp: Ca_10_(PO_4_)_6_(OH)_2_) is a type of calcium phosphate with the Ca/P ratio of 1.67, and is the most stable in the body as well as a major inorganic component of bones and teeth. The crystal structure of HAp belongs to the hexagonal system (space group P6_3_/m), with lattice constants of *a* = 0.9422 nm and *c* = 0.6883 nm. The crystal structure of HAp is shown in Figure 5. The four columnar Ca are aligned parallel to the *c*-axis and the six screw axis Ca surround the *c* -axis at the four corners of the unit cell. Moreover, the hydroxyl groups exist in the part surrounded by the screw Ca [83]. It is well known that HAp has biocompatibility, bone affinity, high absorptivity with biomolecules, and ion exchange properties. Therefore, HAp as a biomaterial has been widely studied [84,85]. When synthetic HAp is implanted as artificial bone in a bone defect, the artificial bone is gradually replaced with newly formed bone tissues under the synergistic action of osteoclasts and osteoblasts to achieve the purpose of bone regeneration [86,87]. However, this process has a high cost over a long period of time. The different cations can be substituted for calcium ion sites, and the different anions can be substituted for phosphate group and hydroxy group sites of HAp. In fact, calcium ions are replaced with iron ions, magnesium ions, and strontium ions, and phosphate groups and hydroxide ions are replaced with fluorine ions and carbonate ions in bones and teeth. For example, fluoride toothpaste has been used to enhance the acid resistance of teeth and prevent cavities [88].

Both octacalcium phosphate (OCP: Ca_8_H_2_(PO_4_)_6_·5H_2_O) and dicalcium phosphate dihydrate (Brushite, DCPD: CaHPO_4_·2H_2_O) have been presumed as the possible precursors to the formation of HAp [89]. The phase transformation from OCP and DCPD to HAp is closely dependent on the supersaturation, the pH of the solutions, and the presence of foreign ions in the biomimetic synthesis process [90]. The HAp is preferentially formed under neutral or alkaline conditions. In acidic solutions, OCP and DCPD phases are often found. The structure of OCP is composed of apatite layers stacked alternately with hydrated layers [91]. It has been confirmed that the transformation of OCP to HAp occurred in neutral solutions such as simulated body fluid through a biomimetic approach, and the presence of Mg^2+^ in the solution has been found to inhibit the transformation by interrupting the precipitation process of HAp [92]. On the other hand, the process of DCPD transformation to HAp in aqueous body fluid such as Hank’s balanced salt solution was investigated, and indicated that brushite modified by potassium ions showed faster transformation to HAp than the normal DCPD [93].

### 3.4. Precipitation of Hydroxyapatite on the Assembly, and Their Interfacial Inorganic–Organic Composite Interactions

**Figure 6:**
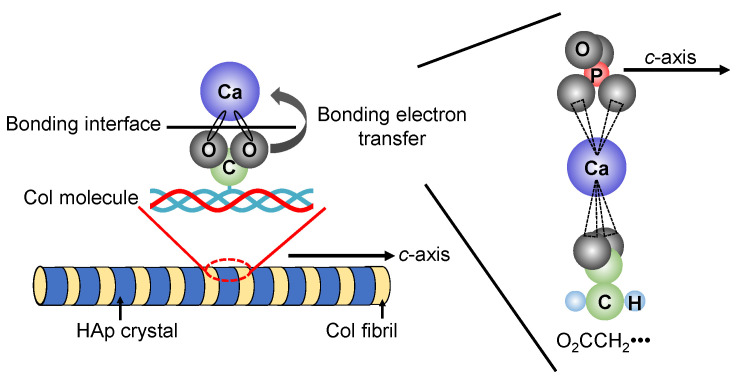
Possible interfacial state between HAp and Col.

In recent years, research has been actively conducted to create functional materials that are similar to the structure of bone tissues by imitating the precipitation process of HAp crystals on Col assemblies in vivo. J.C. Góes et al. precipitated HAp on the Col film surface by alternately immersing the Col film with various different densities of carboxyl groups in CaCl_2_ aqueous solution and K_2_HPO_4_ aqueous solution for 100 cycles [94]. Their results suggested that the higher content of carboxyl groups in the Col film plays an effective role in the heterogeneous nucleation of apatite. In addition, the illustration of the interfacial state between HAp and Col was shown in Figure 6. The chemical bond is formed between the carboxyl group (-COO^−^) protruding vertically from the Col molecule and the calcium ion (Ca^2+^) present on the surface of HAp. In the carboxyl group, two oxygen bonds are bonded to one carbon in the center, and the sigma bonds are formed between the carbon atom and the two oxygen atoms. Furthermore, since the carboxyl group has a valence of −1, one electron *e*− spreads over the entire O-C-O bond to form the π bond.

### 3.5. Current and Possible Applications for Bone Tissue Regeneration and Therapy

**Figure 7:**
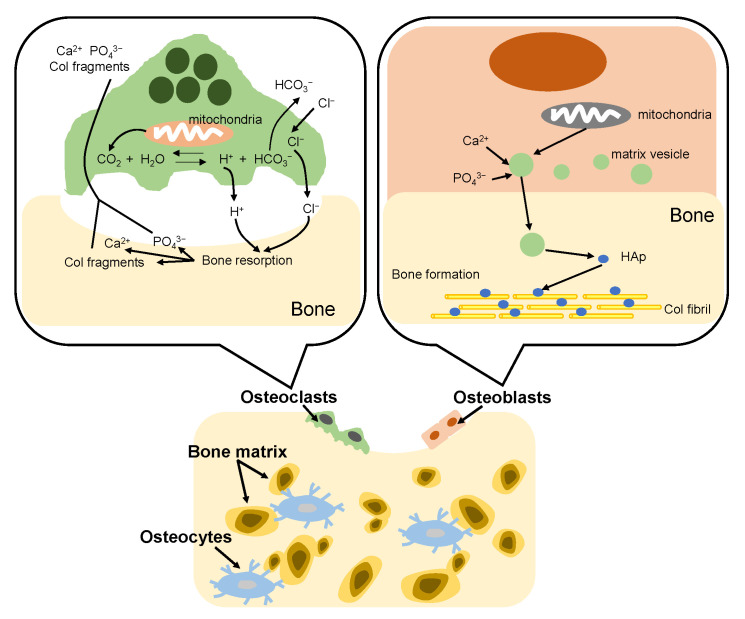
Illustration of bone remodeling.

The bone formation process in vivo is called bone remodeling. The remodeling consists of two complementary processes: bone resorption and bone formation [95], as shown in Figure 7. Adult bone remodeling proceeds in an order such that osteoclasts first adsorb bone, and then osteoblasts form bone. Osteoclasts are found on surfaces of bone. When osteoclasts adhere to bone, a local space is formed between the cells and the bone. Osteoclasts release acid into the local space and thus create an acidic microenvironment, which increases solubility of bone mineral [96]. As a result, the bone mineral (HAp) dissolves into calcium and phosphate ions. Meanwhile, osteoclasts release Col-degrading enzymes which can decompose and absorb the Col fibrils, which become Col fragments. On the other hand, osteoblasts firstly synthesize Col molecules inside the cell and release them out of the cell and the Col molecules spontaneously line up to form fibrils. Then, osteoblasts mediate bone mineralization [97]. Osteoblast matrix vesicles concentrate calcium and phosphate ions from the cytosol and mitochondria, and transfer to the newly formed Col matrix. Subsequently, the matrix vesicles continue to accumulate calcium and phosphate ions from the ion-rich environment until precipitation occurs. The newly formed HAp crystals are precipitated on the Col fibrils, providing nucleation sites for continued crystal growth. In bone tissue, remodeling is constantly occurring due to the bone formation of osteoblasts and the bone resorption of osteoclasts. However, the defects of bone are difficult to self-repair when many parts of the bone are excised due to bone cancer etc. Therefore, a material that fills the defect site and promotes regeneration of the surrounding bone tissue is required.

Large segmental bone fractures cannot be repaired naturally and orthopedic surgery is necessary. Bone defects are currently cured using bone graft materials with biocompatible properties. There are many bone graft materials such as bioceramics [98], biopolymers [99] and organic-inorganic composites [100] which are used for promoting regeneration of the surrounding bone tissue. However, the above bone graft materials readily lead to nerve injury, infections, morbidity and chronic pain. Therefore, the technology to fabricate oriented collagen fibril arrays and collagen/hydroxyapatite composite nanostructures similar to biological tissues is necessary.

## 4. Oriented Mesoporous Silica Films

### 4.1. General Synthesis and Characteristics of Mesoporous Silica Films

**Figure 8:**
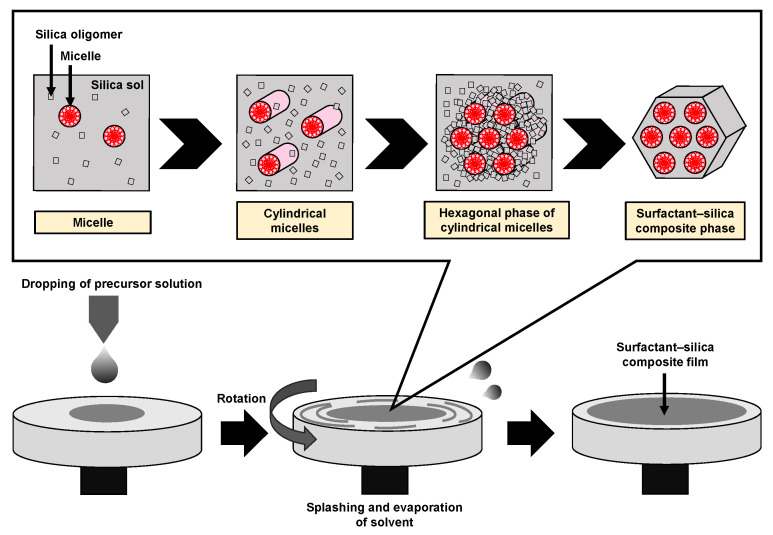
Illustration of the formation of the surfactant–silica composite film in the spin-coating process.

**Figure 9:**
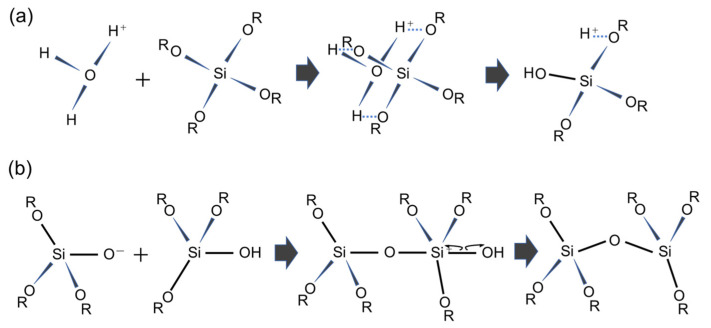
Acid-catalyzed sol–gel process of TEOS. The reaction formula of (**a**) the hydrolysis mechanism and (**b**) the dehydration mechanism.

**Figure 10:**
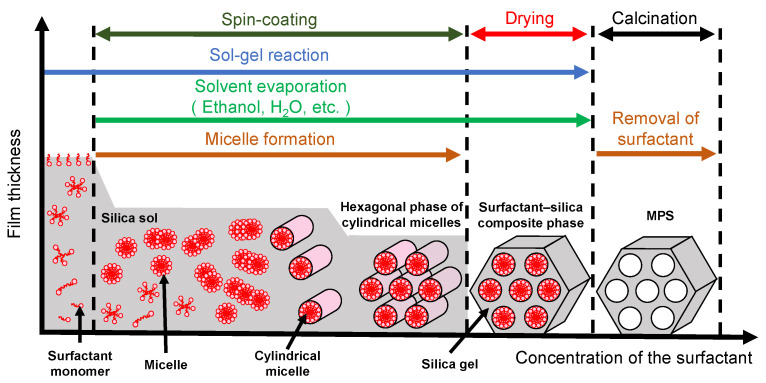
Illustration of the surfactant-silica composite phase changing with the concentration of the surfactant in the spin-coating and calcination processes.

Mesoporous silica (MPS) has uniform pore sizes in the range of 2 to 50 nm, large specific surface areas, high stability, and biocompatibility. The MPS materials have been widely synthesized and applied in the biomedical fields, such as drug delivery [101,102,103], diagnosis [104,105,106], and bone repair [107,108], indicating that MPS can safely exist in our body. MPS can be easily functionalized by different chemical functional groups or proteins to improve its biocompatibility and promote cell adhesion without denaturation [109,110,111]. The pore sizes, surface areas, and arrangement of pore structures can be controlled by the different surfactant types and concentrations [112,113,114]. Both the particle shapes and filmed states of MPS can be synthetized by utilizing the sol–gel method. In detail, the precursor solution consisting of the surfactant, silicon alkoxide (tetraethyl orthosilicate (TEOS), etc.), acid catalyst, and solvent (water, ethanol, etc.) is cast on the arbitrary substrate and then is spin-coated to synthesize the MPS films [115]. As shown in Figure 8, the surfactant molecules could be self-assembled into different micelle phases as a result of an increase in the concentration of surfactant due to splashing and evaporation of solvent by rotation. Simultaneously, the hydrophilic groups of micelles electrostatically interacted with the silica oligomers which were prepared by the hydrolysis and condensation reactions of silicon alkoxide during the sol–gel process, resulting in the formation of a surfactant–silica composite film. The formation of the silica oligomers is caused by the growth of the siloxane network due to the sol–gel reaction of the silicon alkoxide. The mole fractions of acid catalyst and H_2_O are important factors that govern the formation of the siloxane network and the speed of the sol–gel reaction. As shown in Figure 9, there is a sol–gel reaction mechanism using the acid catalyst. In the hydrolysis process, the oxygen of the alkoxy group is attacked by the electrophilic reaction of H_3_O^+^, producing a hydroxy group and alcohol as by-products (Figure 9a). Then, the deprotonated silanol group forms the siloxane bond by nucleophilically attacking the Si atom in the dehydration condensation process (Figure 9b) [116]. Subsequently, the transition of the micelle phases and the siloxane network growth collaboratively occur in the spin-coating and calcination processes, as shown in Figure 10. The thickness of the film in the spin-coating process gradually decreases leading to an increase in the concentration of surfactant. The surfactant monomers would be self-assembled to be the micelles when the concentration of the surfactant monomers exceeds a critical micelle concentration. Subsequently, the micelle phases are changed to be cylindrical micelles and the hexagonal phase of the cylindrical micelles by increasing the concentration of surfactant. At the same time, the polymerization of silica oligomers occurs due to the growth of the siloxane network. After that, the surfactant–silica composite phase is formed with the drying process, and the MPS film is prepared by the calcination process through the removal of surfactant micelles.

### 4.2. Various Techniques for Orientation

There are molecular crystals (hydrated crystals) formed with water molecules and other polar solvents in many ionic surfactants and some nonionic surfactant systems at low temperatures [117,118,119]. The hydrated crystals melt at temperatures above the Krafft point and form molecular assemblies of surfactants such as micelles and lyotropic liquid crystal phases. The surfactant molecules self-assemble to be micelles at the temperature above the Krafft point and the concentration above the critical micelle concentration, and further become lyotropic liquid crystal phases such as hexagonal and lamellar liquid crystal phases at higher concentrations. The lyotropic liquid crystal phase is involved in the formation of the mesostructured MPS, and the interaction between the liquid crystal phase and the surface of the substrate determines the crystallographic orientation of the mesostructure with respect to the substrate. The crystallographic orientation of the MPS film in the out-of-plane direction on the substrate surface is often uniquely determined. However, the in-plane orientation of the film is not controlled, especially on isotropic substrates such as glass. As a result, in the case of a cylindrical upper micelle, the cylinder has a winding structure in the plane; and in the case of a spherical micelle, a plurality of domains having different orientations in the plane are formed. In-plane orientation control of the MPS film is roughly achieved by two methods. One is a method using an external field. For example, in one method, the microcapillaries and an electric field were used to guide the growth direction of the surfactant–silica composite mesostructure [120]. Here, an electric field applied in the direction of the microcapillaries induced an electroosmotic flow of the MPS precursor solution along the microcapillaries, and the rate of silica polymerization was promoted due to the generation of localized Joule heating. Another method uses a substrate with structural anisotropy on the surface. For example, it is reported that an MPS film was formed on silicon substrates with different crystal orientations by hydrothermal synthesis, and the orientation of MPS mesochannels was controlled in one direction on the (110) plane with strong anisotropy of atomic arrangement [121]. However, the above methods are not suitable to synthesize the MPS film with in-plane orientation on isotropic substrates such as glass. Thus, it is necessary to develop a technology to control the in-plane orientation of the MPS film.

### 4.3. Possibility for Biological and Medical Applications

There are few reports on the synthesis of MPS films for biomedical applications apart from our research group. According to our previous study [122], osteoblast-like cells were cultured on the MPS film, indicating that the high adhesion density of cells and formation of bone tissue were promoted. The MPS films with high specific surface area are suitable for adsorbing proteins and further promoting cell adhesion. However, the in-plane orientation of the MPS films was not successfully controlled, resulting in randomly aligned morphologies of the adsorbed cells. Thus, the MPS films with in-plane orientation have the potential to guide the anisotropic shape of the adsorbed proteins and further control the direction of cell adhesion and growth. Therefore, the oriented MPS films show excellent properties for biomedical applications, such as cell culture and surface modification of biomaterials.

## 5. Rubbing-Treated Polyimide Film for Biological and Medical Applications

### 5.1. Synthesis and Characteristics

**Figure 11:**
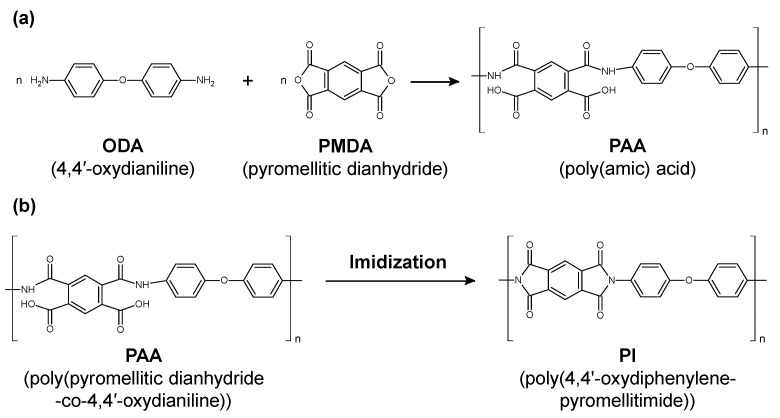
(**a**) Chemical structural change from poly(amic acid) to polyimide (PI). (**b**) Representative change from poly(pyromellitic dianhydride-co-4,4′-oxydianiline) to poly(4,4’-oxydiphenylene-pyromellitimide) through baking.

Polyimide (PI) as a polymer material has been used for medical catheters because of its high flexibility, mechanical strength, and chemical resistance [123,124]. PI is classified into several types according to the functional groups (aromatic or aliphatic) and side chains contained in the main chain of the polymer. A common technique in PI synthesis is a two-step synthetic process mediated by poly(amic acid) (PAA), as shown in Figure 11. First, aromatic dianhydride and aromatic diamine are reacted in a polar solvent to synthesize an easily soluble polyamic acid, which is then heat imidized to obtain PI. The PAA is made from 4,4’-oxydianiline (ODA) and pyromellitic dianhydride (PMDA) (Figure 11a). The PI was synthesized via the dehydration of PAA and imide ring formation by thermal cross-linking (Figure 11b). It has been reported that the imidization ratio of (PMDA-ODA) PI is affected by the baking temperature, where the imidization was fully completed at the baking temperature of 220 °C [125].

### 5.2. Practical Situations and Problems

**Figure 12:**
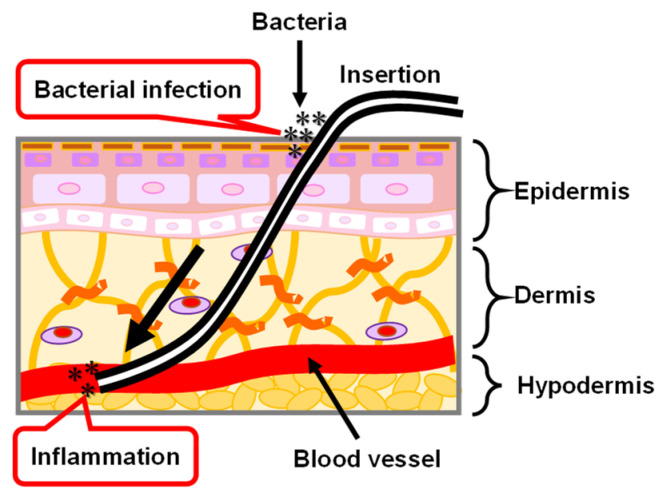
Illustration of skin structure and the problem of PI catheter applied in the biomedical application.

The medical PI catheter is directly connected to the internal and external tissues of the human body by passing through the skin. As shown in Figure 12, the skin is roughly divided into three layers that are the epidermis, dermis, and hypodermis tissues, from the outside to the inside [126,127,128]. Epidermal healing plays an important role in the repair of the wounded skin tissue because all wounds are eventually covered by epithelium. In the epidermis, keratinocytes, the main cells that make up 95% of the epidermis, are highly organized and arrayed. The keratinocytes are divided in the basal layer and subsequently move to the stratum corneum which is the outermost layer of the epidermis. In the stratum corneum, keratin filaments—which are structural proteins—align parallel to the plane of the flattened stratum corneum cells. When the PI catheter passes through the skin tissues, the interface between the PI catheter and skin tissue cannot be tightly combined because of foreign body reactions [129], resulting in skin tissue being exposed to air. Thus, there is a risk of bacterial infection and inflammation. The possible reason for the foreign body reaction between the PI catheter and skin tissues is that the proteins are randomly adsorbed on the PI catheter surface. The morphology of the adsorbed proteins on the PI catheter surface are different from the regularly oriented proteins in vivo. Therefore, a coating technology for the PI catheter is necessary by using an oriented biocompatible material such as MPS which has the potential to control the orientation of adsorbed proteins and to further encourage the repair of the skin tissues.

### 5.3. Rubbing Treatment Techniques

**Figure 13:**
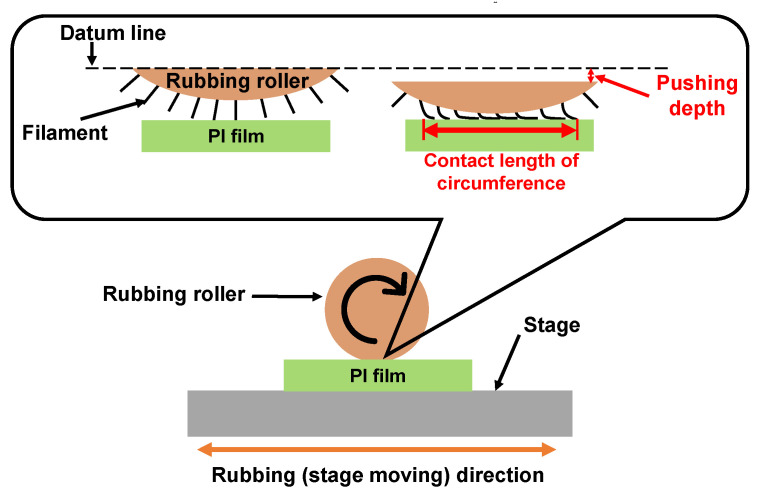
Illustration of the rubbing treatment process for PI film.

Rubbing treatment is a technique by which the PI film surfaces are rubbed in one direction using cloths such as rayon, cotton, etc., as shown in Figure 13. In the rubbing process, the radius of the rubbing roller, the rotation speed, the number of rubbing times, the contact length of circumference with the substrate, the moving speed of the rubbing stage, etc. are parameters related to the strength of rubbing. The contact length of circumference is a parameter related to the pushing depth. The control point is decided when the contact distance between the filament top and the substrate surface is 0. Thus, the contact length of circumference increases with increasing contact length. The strength of the rubbing was formulated by [130] as Equations (1) and (2) below [130]. In Equation (1), *D* is a function of rubbing density; *γ* is a function of rubbing pressure, filament density and friction coefficient; and *L* is the total length of the rubbing cloth that contacts a certain point of the substrate. However, since γ is a function caused by the difference in friction coefficient depending on the material, it is difficult to determine an accurate value. Therefore, the rubbing strength is defined to be *L* when the rubbing cloth is fixed, as shown in Equation (2). *N* is the number of rubbing times; *l* is the contact length of circumference; *r* is the radius of the rubbing roller; *n* is the rotation speed of the rubbing roller; and *v* is the moving speed of the rubbing stage.

*D* = *γ* × *L* (1)

*L* = *N* × *l* (1 + 2π*rn*/60*v*) (2)

### 5.4. Film Formation Mechanisms on Rubbing-Treated Film

Rubbed PI films as a polymer film with structural anisotropy have been widely used to control the orientation of liquid crystals [131,132,133]. However, the orientation mechanism of rubbing treatment is not fully understood. There are several hypotheses that have been proposed [134,135,136]. The general hypothesis is that the grooves along the rubbing direction are formed by mechanical contact between the PI film and rubbing cloth. The groove-based liquid crystal orientation theory proposes an orientation mechanism based on geometric effects caused by elastic strain energy. Specifically, the theory is that the driving force works to make the elastic strain energy to be minimum, leading to the long axis of the liquid crystal molecule being oriented parallel to the grooves. On the other hand, the other hypothesis is that there is a stretching-orientation effect of the polymer main chain due to friction. The extension direction of the polymer main chain is parallel to the rubbing direction due to the generation of stress and frictional heat during the rubbing treatment, and the oriented polymer main chains interact with the conformation of the liquid crystal. As a result, the orientation direction of the polyimide main chain in the rubbed film and the inclination direction of the pretilt of the liquid crystal are the same. In addition, the rubbed PI films have also been applied to control the orientation of copper phthalocyanine [137] and copolymer [138]. The rubbing treatment technique has the characteristics of low cost and easy operation, and the oriented nanostructures of materials could be effectively controlled by utilizing the physicochemical anisotropy of the surface of the rubbed PI film. However, it has not been applied in the biomedical field for fabricating oriented nanobiomaterials. Thus, it is necessary to investigate the interactions between nanobiomaterials and rubbed PI films and to further develop the fabrication of one-directional nanobiomaterials for wide application in the biomedical field.

### 5.5. Biological and Medical Applications

The oriented nanostructure in biological tissues is extremely important in determining the function of the tissue. However, no fabrication technology for highly-oriented nanostructures similar to biological tissues has previously been established. Therefore, an effective technology was developed to fabricate highly-oriented nanobiomaterials in our group. Here, we mainly introduce the reports regarding the fabrication of highly-oriented nanobiomaterials by utilizing the physicochemical anisotropy of the surface of rubbed PI film. The orientation of surface functional groups and PI main chains of the PI film was effectively controlled by rubbing treatment to obtain the rubbed PI film. For example, highly-oriented homogeneous Col fibril arrays were successfully fabricated on the rubbed PI film for precipitation of CP crystals [139,140]. The mechanism of highly-oriented Col fibril arrays on the rubbed PI films was investigated in order to understand the interfacial interactions. It was found that the orientation of surface functional groups and nano-grooves of the rubbed PI film was effectively controlled. The highly-oriented Col fibrils were formed inside the nano-grooves by the formation of hydrogen bonds between the C=O of the imide groups (rubbed PI film) and the N–H of the amino groups (β-Sheet of Col molecules), resulting in the Col molecules being oriented parallel to the rubbing direction and subsequently being self-assembled into fibrils. Thus, the orientation and density of the fibril arrays on the films were successfully controlled by the interfacial interactions between the β-Sheet component of Col and the surface nano-grooves of the rubbed PI films. Moreover, the CP crystals were precipitated and grown along the Col fibrils after immersion into a simulated body fluid. The highly-oriented CP/Col fibril hybrid nanostructures were similar to bone tissues and have the potential to be applied as bone graft materials for the regeneration and repair of defective bone tissue.

On the other hand, highly-oriented cylindrical MPS films were synthesized on rubbed PI film by adjusting the molar ratio of the orientation-directing agent (Brij56) to the structure-directing agent (P123) as surfactants in the silica precursor solutions for guiding protein adsorption states [141]. In detail, the micro-grooves were formed and PI main chains were oriented on the surface of the rubbed PI films. The semi-cylindrical Brij56 micelles were oriented in the direction perpendicular to the rubbing direction by the strong hydrophobic interaction between the alkyl groups of Brij56 (‒C_16_H_33_) and aromatic rings of the PI main chain. Then, the spherical P123 micelles also transformed into cylindrical micelles that were laminated on the semi-cylindrical brij56 micelles, resulting in the hierarchical growth of cylindrical P123 micelles. Simultaneously, the hydrophilic groups of micelles electrostatically interacted with the silica oligomers which were prepared by the hydrolysis and condensation reactions of silicon alkoxide during the sol–gel process, resulting in the transition of the micelle phases and the siloxane network growth collaboratively occurring to form the mesostructured surfactant–silica composite film. After that, the highly-oriented cylindrical MPS film was prepared by the calcination process through the removal of surfactant micelles. Moreover, the proteins with the anisotropic adsorption morphologies were adsorbed on the highly-oriented cylindrical MPS film. Thus, the synthesized highly-oriented cylindrical MPS film is useful to control the anisotropic adsorption shapes of the mesoscale biomolecules such as proteins and to further encourage oriented-cell adhesion. Therefore, it has the potential to be used as a new coating technology for the biomedical PI catheter.

**Figure 14:**
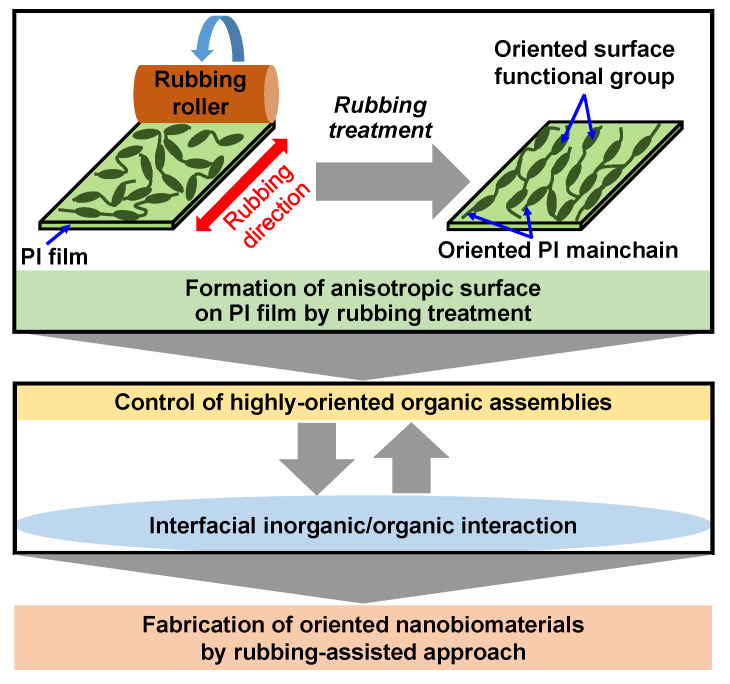
Scheme of rubbing-assisted approach for fabricating oriented nanobiomaterials.

According to the above results, a rubbing-assisted approach for fabricating highly-oriented inorganic/organic composite nanobiomaterials was developed, as shown in Figure 14. The orientation of surface functional groups and PI main chains of the PI film was effectively controlled by rubbing treatment to obtain the rubbed PI film with an anisotropic surface. The highly-oriented organic assemblies such as Col fibrils, surfactant micelles, etc. can be effectively controlled on the rubbed film. Furthermore, the highly-oriented inorganic/organic composite nanobiomaterials were easy to be further fabricated by interfacial inorganic/organic composite interaction in the biomimetic process. Therefore, the rubbing-assisted approach is an effective technology to fabricate one-directional inorganic/organic nanobiomaterials, indicating the potential for wide application in the biomedical field.

## 6. Conclusions

The oriented structures in biological tissues such as cornea, bone, diatoms, etc., were reviewed, and the techniques for producing highly-oriented nanobiomaterials by imitating the oriented organic/inorganic nanocomposite mechanism of biological tissues were summarized. In particular, we introduced a fabrication technology for obtaining the highly-oriented structure of biomaterials on the surface of a rubbed PI film that has physicochemical anisotropy in order to further form the highly-oriented organic/inorganic nanocomposite structures based on interface interaction. The highly-oriented homogeneous Col fibril arrays fabricated on the rubbed PI film for precipitating CP crystals and the highly-oriented cylindrical MPS films synthesized on the rubbed PI films for guiding anisotropic adsorption states of proteins were introduced. It was indicated that the highly-oriented nanobiomaterials fabricated by the above biomimetic process could be applied in the biomedical field.

## Data Availability

Not applicable.
